# Screen Time Parenting Practices and Associations with Preschool Children’s TV Viewing and Weight-Related Outcomes

**DOI:** 10.3390/ijerph18147359

**Published:** 2021-07-09

**Authors:** Cody D. Neshteruk, Gina L. Tripicchio, Stephanie Lobaugh, Amber E. Vaughn, Courtney T. Luecking, Stephanie Mazzucca, Dianne S. Ward

**Affiliations:** 1 Department of Population Health Sciences, School of Medicine, Duke University, 200 Morris Street, Durham, NC 27701, USA; 2 Department of Social and Behavioral Sciences, Temple University, Philadelphia, PA 19122, USA; gina.tripicchio@temple.edu; 3 Department of Epidemiology and Biostatistics, Memorial Sloan Kettering Cancer Center, New York, NY 10065, USA; stephanie.lobaugh0630@gmail.com; 4 Center for Health Promotion and Disease Prevention, University of North Carolina at Chapel Hill, Chapel Hill, NC 27599, USA; a.vaughn8@icloud.com (A.E.V.); dsward@email.unc.edu (D.S.W.); 5 Department of Dietetics and Human Nutrition, University of Kentucky, Lexington, KY 40506, USA; Courtney.Luecking@uky.edu; 6 Prevention Research Center, Washington University in St. Louis, St. Louis, MO 63130, USA; smazzucca@wustl.edu; 7 Department of Nutrition, University of North Carolina at Chapel Hill, Chapel Hill, NC 27599, USA

**Keywords:** screen time, screen media, parenting practices, obesity, preschool children

## Abstract

The purpose of this study was to examine associations between screen time (ST) parenting practices and 2–5-year-old children’s TV viewing and weight status. Data were collected from 252 parent–child dyads enrolled in a randomized parent-focused childhood obesity prevention trial from 2009–2012. ST parenting practices were assessed at baseline using a validated parent-reported survey. Parent-reported child TV viewing and objectively measured anthropometrics were assessed at baseline, post-intervention (35 weeks), and follow-up (59 weeks). Marginal effect models were developed to test the association between baseline ST parenting practices and children’s TV viewing, BMI z-score, and waist circumference across all time points. Limiting/monitoring ST was associated with decreased weekly TV viewing (β = −1.79, 95% CI: −2.61; −0.95), while exposure to TV was associated with more weekly TV viewing over 59 weeks (β = 1.23, 95% CI: 0.71; 1.75). Greater parent use of ST as a reward was associated with increased child BMI z-score (β = 0.15, 95% CI: 0.03; 0.27), while limiting/monitoring ST was associated with decreased BMI z-score (β = −0.16, 95% CI: −0.30; −0.01) and smaller waist circumference (β = −0.55, 95% CI: −1.04; −0.06) over the study period. These findings suggest that modifying parent ST practices may be an important strategy to reduce ST and promote healthy weight in young children.

## 1. Introduction

In the United States, more than one in three children have overweight or obesity [1]. Modifying children’s screen time (ST) or media use (i.e., time spent watching television (TV), playing video games, or using computers, tablets, and mobile phones) may be one strategy to combat the rise in pediatric obesity [2]. Both observational and experimental research has demonstrated a clear link between ST and children’s weight gain and adiposity [3]. As such, the American Academy of Pediatrics (AAP) recommends that ST should be limited to one hour a day of high-quality programming for 2–5-year-old children [4]. However, many children exceed this recommended limit [5,6,7]. Furthermore, ST behaviors established in childhood tend to track into adolescence [8], indicating a need to identify strategies that can effectively reduce young children’s ST and promote healthier weight status.

Parents influence children’s ST through their ST parenting practices, which are specific behaviors performed by parents that shape their child’s attitudes, beliefs, or behaviors related to ST or media use (e.g., co-viewing, setting rules or limits on ST, monitoring ST) [9]. Much of the research on parent ST practices has been cross-sectional and focused on the impact of rules and limits on the time children spend viewing TV, with findings indicating mixed results [10,11]. Other cross-sectional studies have examined the effect of parent ST practices on daily physical activity outcomes, showing that ST practices tend to reduce overall physical activity and increase total sedentary behavior [12,13]. Very few studies have investigated the direct association between ST practices and children’s weight, especially longitudinally. Mihrshani et al. showed that in a sample of Australian children, adolescents had a higher odds of overweight and obesity when their parents did not have any rules around screen time, while Sleddens et al. found that restriction of sedentary behavior at age five was associated with child BMI z-score at 7 in a cohort of Dutch children [14,15]. However, there is a dearth of literature examining associations in young children, when prevention efforts can have greatest impact. Therefore, the aims of this study were to examine the associations between parents’ ST practices and children’s TV viewing time as well as weight outcomes over the course of approximately one year. Based on existing evidence and research in other parenting domains (i.e., food and physical activity), we hypothesized that ST practices creating structure (e.g., setting limits and monitoring children’s ST) would be associated with decreased TV viewing time and weight in children, while practices that were overly controlling or promoting of ST (e.g., using ST as a reward) would be associated with increased TV viewing time and weight. 

## 2. Methods

This study used longitudinal data from the Parenting SOS study conducted from 2009–2012. Parenting SOS was a randomized controlled trial testing a 35-week parent-focused childhood obesity prevention intervention compared to a child literacy control (Clinical Trials ID: NCT 00998348) [16]. This dataset provided data on parent–child dyads at three time points: baseline (*n* = 252), post-intervention (35 weeks, *n* = 208), and follow-up/maintenance (59 weeks, *n* = 181). All protocols were approved by the University of North Carolina at Chapel Hill Institutional Review Board. 

### 2.1. Recruitment

Recruitment procedures have been described elsewhere [16]. Briefly, families were recruited from central North Carolina using a variety of different recruitment methods including: direct mailings, listserv announcements, advertisements in the community, and interception at child care centers. To be eligible, families had to have at least one child between the ages of 2–5 years, at least one parent with overweight or obesity (body mass index (BMI) ≥ 25 kg/m^2^) based on self-reported height and weight, and be able to speak and read English. The parent with overweight or obesity did not have to be the parent who completed measures, and there was no inclusion or exclusion criteria specified based on parent gender or household/family socioeconomic status.

### 2.2. Data Collection

At each time point (baseline, post-intervention, follow-up), families attended in-person measurement events at convenient community locations. During these events, parents completed a series of questionnaires about family demographics, ST parenting practices, and child TV viewing time. Anthropometric measures including parent and child height and weight and child waist circumference were objectively measured. Informed consent was obtained from all participants at the beginning of the baseline measurement event.

### 2.3. Measures

Family demographics. Parent baseline surveys included a demographic questionnaire that captured parent sex, age, race/ethnicity, marital status, education, annual family income, and employment status. This survey also captured child sex and date of birth.

ST parenting practices. Parents’ ST parenting practices were measured at each time point using a self-report questionnaire developed specifically for Parenting SOS [17]. Items assessed parents’ practices using Likert responses (e.g., never to very often; strongly disagree to strongly agree) and open ended questions (e.g., “About how much time is s(he) allowed to watch TV, videos or movies each weekday”). A list of the items is available in Supplementary File S1. Items were reverse coded when necessary to ensure that responses reflected greater use of the practice and open-ended questions were categorized. Previous exploratory factor analyses with the baseline data identified four ST parenting practice subscales: (1) limiting/monitoring ST (10 items), (2) use of ST to reward/control behavior (4 items), (3) exposure to TV (3 items), and (4) explicit modeling/enjoyment of ST (6 items) [17]. Subscale scores were calculated by averaging the responses to each item within the subscale, with higher scores indicating greater use of the ST practice. Sample items, the possible range of scores, and internal consistency for each ST practice subscale are shown in Table 1. All four subscales demonstrated acceptable reliability and construct validity in the original analyses [17].

Child TV viewing. Children’s weekly TV viewing was reported by parents at each time point using two items. Parents were first asked to report the total hours that the child spent watching TV, videos, or movies for the previous five weekdays (item 1) and then the total hours for the previous two weekend days (item 2). Responses from these two items were summed together to yield children’s total hours of weekly TV viewing.

Parent and child anthropometrics. Parent and child anthropometrics were measured at each time point by trained data collectors. Standing height was measured to the nearest 1/8 inch using Shorr or Seca infant/child/adult stadiometers (Shorr Productions, Olney, MD; Seca Corporation, Columbia, MD) and weight to the nearest 0.1 pound with a Seca model 770 portable electronic scale (Seca Corporation, Columbia, MD). Child waist circumference was measured to the nearest 0.1 cm using a Gulick II measuring tape. All measures were taken at least twice to ensure accuracy and then averaged. Height and weight were used to calculate parent BMI. Child BMI z-score was calculated using age-and-sex-specific SAS code from the Centers for Disease Control and Prevention [18].

### 2.4. Analyses

All analyses were conducted in SAS version 9.4 (Cary, North Carolina). Descriptive statistics (i.e., means and frequencies) were calculated to describe the sample characteristics. Marginal effect models were constructed to examine the association between parents’ baseline ST parenting practices and children’s weekly TV viewing, child BMI z-score, and child waist circumference using data from each of the three time points. Only baseline ST parenting practices were used in these models, as their repeated measurement showed little to no change in scores over time. The MIXED procedure was used to generate repeated measures marginal effect models to test the association between screen time parenting practices and child outcomes. An unstructured covariance structure was used to account for the correlation between repeated observations from the same child. This approach also accounts for attrition across the three time points, allowing for the utilization of all available data and accounting for data believed to be missing at random. Lower-income families were more likely to be lost to follow-up, so family income was included as a covariate in all models. Randomization group was also included in all models to account for any potential intervention effect. Other covariates including time, parent BMI, and child sex, age, and BMI z-score were included. A significance level of α = 0.05 was used.

## 3. Results

Baseline parent demographic characteristics are shown in Table 2. Almost all parent respondents were mothers (94%). The majority of parents were either white (55%) or African American (34%), married/living with a partner (80%), and had a college or graduate level education (83%). Annual family income was generally above USD 50,000 per year (69%), and most parents worked full-time jobs (64%). Half the children were female (50%), and the average age of children was 3.5 (±0.8) years. Baseline measures showed that children spent an average of 12.6 (±9.5) hours per week watching TV, with 67% of children exceeding the AAP recommendation of one hour of ST per day. Most parent respondents had overweight or obesity (71%) with a mean BMI of 29.5, while most children had a healthy weight (69%) with a mean BMI z-score of 0.34.

Associations between each of the four ST parenting practices and child outcomes are shown in Table 3. The population-averaged parameter estimates from across the three time periods are presented. Limiting/monitoring ST was inversely associated with weekly TV viewing (β = −1.77, 95% CI: −2.61 to −0.94), meaning that for each unit increase in the limiting/monitoring ST score, children watched an additional 1.77 h of TV. Limiting/monitoring ST was also inversely associated with child BMI z-score (β = −0.16, 95% CI: −0.30 to −0.02) and child waist circumference (β = −0.56, 95% CI: −1.05 to −0.07). Using ST as a reward was not associated with weekly TV viewing or child waist circumference but was positively associated with child BMI z-score (β = 0.15, 95% CI: 0.03 to 0.27). Parent explicit modeling/enjoyment of ST was not associated with weekly TV viewing, child BMI z-score, or child waist circumference. Exposure to TV was positively associated with weekly TV viewing (β = 1.22, 95% CI: 0.70 to 1.74) but was not associated with child BMI z-score or child waist circumference.

## 4. Discussion

The current study examined the association between ST parenting practices at baseline and young children’s weekly hours of TV viewing, BMI z-score, and waist circumference over a one-year period. As hypothesized, the structure-based practice of limiting/monitoring of ST was associated with reductions in children’s TV viewing, BMI z-score, and waist circumference across the study period. Meanwhile, the controlling practice of using ST to reward/control behavior as well as the ST promoting practice exposure to TV were associated with increased child BMI z-score and more hours of weekly TV viewing, respectively.

Key findings from this study were that greater parental limiting and monitoring of children’s ST was associated with reduced weekly TV viewing, BMI z-score, and waist circumference in children. These results are consistent with findings from other studies in the weight-related parenting literature showing that structure-based practices such as monitoring and rule-setting are associated with positive eating behaviors, improved physical activity, and a reduction in obesity risk [19]. Because limiting and monitoring ST was associated with each outcome of interest, this suggests it may be a potentially influential strategy parents can use to reduce children’s ST and manage children’s weight. Clinical and public health professionals can encourage and assist parents in setting clear limits around the amount of ST. Future studies should further examine type of ST (e.g., TV, video games, smart phone, etc.), content (e.g., educational vs. entertainment), and context for children’s ST (e.g., when, with who, where) to better assist parents in creating structure for their children in the current media environment.

Based on the broader parenting literature, structure-based practices such as limiting and monitoring must include elements of demandingness (control and demands on the child) and responsiveness (warmth and support offered to the child) [20]. In terms of demandingness, clear limits must not only be set, but also enforced. Hence, parents should be prompted to monitor their children’s ST and media use to ensure that these rules are being followed consistently. In terms of responsiveness, limits should be set in such a way that foster individuality and self-regulation. Hence, parents should be encouraged to work with their children to set these rules and limits (e.g., when children can use media or what type of media device children can use), giving children a sense of control and potentially making it more likely that they will follow family rules and limits around ST. Guidance around these practices should be adapted to the specific constraints of the family, recognizing that monitoring may not always be possible or that rules and limits may look different for different families based on family circumstance (e.g., parent work schedule may make it difficult to monitor child ST).

Another important finding from this study was that greater use of ST to reward or control children’s behavior was associated with increased child BMI z-score. A cross-sectional study of young children found that mothers’ use of screens to control behavior was positively associated with weekday ST, supporting findings from this study [21]. However, this practice has largely been understudied relative to other ST parenting practices. More generally, controlling practices around weight-related behaviors, such as the use of rewards or bribes, are generally associated with an increase in child weight status [22,23]. For instance, using food as a reward has been associated with an increase in young children’s BMI z-scores over time [24]. The practice of using rewards to help control children’s behavior tends to be a common strategy employed by parents with young children [25]. In fact, using rewards is a recommended parenting practice in some contexts as an effective strategy for managing child behaviors [26]. However, it is necessary to continue to explore the effect of rewarding with ST as it relates to child outcomes, particularly those influencing risk of overweight/obesity. In the context of ST, the use of ST as a reward could potentially increase children’s preference for media over other activities (e.g., active or creative play). Given that the use of rewards is a common practice, educating parents on age-appropriate rewards that are not screen- or food-based is key. These could be experiences (e.g., trip to the park or library) or tangible items (e.g., play with favorite toy). Recognizing that some parents might still decide to use ST as a reward, there should be clear limits on when, what, and how long children are able to engage with screen media.

This study also found children’s exposure to TV was associated with greater weekly TV viewing. However, the measure of TV exposure did not directly assess if children watched TV, but rather background TV exposure throughout the day and during meals. Most existing literature has examined the direct impact of watching TV during mealtimes, showing a negative impact on children’s diet quality, while also leading to more instances of fussiness or behavioral difficulties during mealtimes [27,28,29]. However, in the United States, young children are exposed to an average of 232 min of background TV per day [30]. Furthermore, research has shown that background TV exposure is associated with detrimental effects on children’s play, children’s cognitive performance, and the quality and quantity of parent–child interaction [31,32,33], suggesting that further exploration into the effect of background TV on children’s weight and weight-related behaviors is necessary. Nevertheless, in order to meet the AAP recommendation of no more than one hour of high-quality ST, parents should seek to reduce both children’s direct and indirect exposure to TV through designating TV-free times and locations, setting rules and limits for when the TV can be on, and modeling healthy TV habits (e.g., turning TV off during meals or when not in use).

This study had several strengths including utilizing multiple measurement time points and several objectively measured weight outcomes. Despite these strengths, there were several limitations. Data for this study come from a randomized controlled trial testing an obesity prevention intervention, which may increase the potential for the intervention effects to confound findings given that we collapsed the intervention and control groups into a single cohort. However, ST was not a primary intervention target, and arm assignment was included as a covariate in all models. There was no significant difference between the groups in TV viewing (*p* = 0.65), while intervention children had a higher BMI z-score (*p* = 0.06) and waist circumference (*p* = 0.04) over the time period. This suggests that an intervention effect had minimal influence on our findings. Additionally, although we used ST practice scales that were psychometrically tested, the data from this study were collected between 2009 and 2012 and focused primarily on TV viewing and video games usage. This does not capture the current screen media environment, marked by technological advances and increased use of devices such as smartphones and tablets. With the now-ubiquitous nature of screen-based devices, it could be hypothesized that the associations identified in this study would be even more pronounced in a current sample. This may be further exacerbated by the COVID-19 pandemic, where we have seen increases in children’s ST use [34]. Given our findings and their implications for obesity risk, this warrants further investigation. Another limitation of this study is the missing data at the second and third time points. However, our analytic methods allowed us to account for this and maximize the data used. Finally, the sample in our study was predominantly mothers from middle to high socioeconomic backgrounds. Our findings may not translate to more diverse families with lower income, single parents or to fathers, as some evidence suggests that parent practices may differ among different racial and ethnic groups and between mothers and fathers [35,36,37].

## 5. Conclusions

Results from this study generally confirmed findings from previous studies and demonstrated an association between ST parenting practices and children’s TV viewing and weight status over time in young children aged 2–5 years. These results can help inform pediatric obesity prevention and treatment strategies as well as to inform guidance given to parents about their children’s ST or media use. Specifically, practitioners and researchers should consider parents’ ST parenting practices as a target for obesity prevention in both clinical and research settings. Modifying parents’ ST practices may be one way to effectively reduce children’s ST and positively influence children’s weight status. However, further research is still needed to investigate these associations as the ST environment continues to evolve and to examine whether different types of content (e.g., educational, active media, recreational) have differential effects on children’s weight. Additionally, more work is needed to understand the association between both mothers’ and fathers’ ST practices and children’s screen media usage and weight in more diverse populations.

## Figures and Tables

**Table 1:** Description of ST parenting practice subscales.

Practice (Items)	Sample Items	Mean (SD) ^a^	Possible Range ^b^	Cronbach’s α ^a^
Limiting/monitoring ST (10)	I am in charge of how much TV my child watches during his/her free time at home. I tightly monitor the time my child watches TV or videos/plays video games during the week/weekend. I have control over how much TV my child watches.	3.5 (1.2)	0.5–6.0	0.79
Use of ST to reward/control behavior (4)	How often do you take away TV, video, or movie time a punishment for bad behavior? How often does your child get extra TV, video, or movie time as a reward?	3.1 (1.0)	1.0–5.25	0.79
Exposure to TV (3)	How many days per week does your family have the TV on during breakfast/evening meal? How often is the TV in your house on when people are at home?	2.6 (1.7)	0.33–6.67	0.66
Explicit modeling/enjoyment of ST (6)	I enjoy watching TV/movies with my child. How much do you enjoy watching TV or movies during your free time? During a typical week, how often do you watch TV or videos with your child?	3.8 (0.7)	1.17–5.3	0.76

Abbreviations: screen time (ST), television (TV), standard deviation (SD). ^a^ Calculated using baseline data. ^b^ Scores are an average of all items in that subscale. Not all items used the same response scale yielding fractional minimums and maximums.

**Table 2:** Baseline parent demographic characteristics (*n* = 252).

Parent Characteristics ^a^	*n* (%)
Female	235 (94)
Age, years, mean (SD)	35.6 (5.8)
Race	
White	143 (57.7)
Black	83 (33.5)
Asian/Pacific Islander	8 (3.2)
Other ^b^	14 (5.6)
Hispanic/Latino	14 (5.6)
Marital status	
Married or living with partner	201 (80.1)
Single or never married	36 (14.3)
Divorced or separated	13 (5.2)
Widowed	1 (0.4)
Education	
High school or GED	6 (2.4)
Some college or technical school	37 (14.7)
College	115 (45.8)
Graduate degree	93 (37.1)
Annual family income (USD)	
<25,000	25 (10.3)
25,000–50,000	51 (20.9)
50,000+	168 (68.9)
Employment status	
Full time outside the home	153 (63.8)
Part time outside the home	18 (7.5)
Work at home	17 (7.1)
Stay at home parent	33 (13.8)
Other	19 (7.9)
Parent BMI category ^c^	
Healthy (BMI 18.5–24.9)	73 (29.0)
Overweight (BMI 25–29.9)	80 (31.8)
Obese (BMI ≥ 30)	99 (39.3)

Abbreviations: standard deviation (SD), general education degree (GED), United States dollar (USD), body mass index (BMI). ^a^ Missing: race (*n* = 4); Hispanic (*n* = 2); marital status (*n* = 1); education (*n* = 1); income (*n* = 8); employment (*n* = 12). ^b^ Other includes Native American, multiple races, or another race that was not listed. ^c^ Either parent had to have overweight or obesity, not necessarily the one who completed study measures.

**Table 3:** Association between ST parenting practices and children’s weekly hours of TV viewing, BMI z-score, and waist circumference ^a^.

	Weekly TV Viewing (Hours) ^b^			BMI z-Score ^c^			Waist Circumference (cm) ^d^		
Variables	Estimate (SE)	95% CI	*p* Value	Estimate (SE)	95% CI	*p* Value	Estimate (SE)	95% CI	*p* Value
Limiting/monitoring ST	−1.77 (0.42)	−2.61 to −0.94	<0.001	−0.16 (0.07)	−0.30 to −0.02	0.03	−0.56 (0.25)	−1.05 to −0.07	0.03
ST as a reward	0.61 (0.37)	−0.11 to 1.33	0.10	0.15 (0.06)	0.03 to 0.27	0.02	0.33 (0.22)	−0.10 to 0.76	0.13
Explicit modeling/enjoyment of ST	1.05 (0.65)	−0.23 to 2.33	0.11	−0.07 (0.11)	−0.29 to 0.15	0.55	−0.56 (0.38)	−1.32 to 0.19	0.14
Exposure to TV	1.22 (0.26)	0.70 to 1.74	<0.001	−0.07 (0.05)	−0.16 to 0.02	0.15	−0.24 (0.16)	−0.55 to 0.07	0.12

Abbreviations: screen time (ST), body mass index (BMI), television (TV), standard error (SE), confidence interval (CI). ^a^ Since the population level effect was of interest, the population-averaged parameter estimates from across the time period are presented. ^b^ Model was adjusted for randomization group, family income, child sex, child BMI z-score, child age, and time. ^c^ Model was adjusted for randomization group, family income, parent BMI, and time. ^d^ Model was adjusted for randomization group, family income, child sex, child age, parent BMI, and time.

## Data Availability

Data can be made available by contacting the corresponding author.

## Supplementary Materials

The following are available online at https://www.mdpi.com/article/10.3390/ijerph18147359/s1, File S1. List of items for screen time parenting subscales.
