# Supplementary material for: Screen Time Parenting Practices and Associations with Preschool Children’s TV Viewing and Weight-Related Outcomes
Source: Int J Environ Res Public Health. 2021 Jul 9;18(14):7359. doi: 10.3390/ijerph18147359 (PMC8303526; doi:10.3390/ijerph18147359)
Supplement: Supplementary file 1 [file ijerph-18-07359-s001.zip › ijerph-1257175-supplementary.pdf]

Supplementary File S1. List of items for screen time parenting

subscales Limiting/monitoring of screen time; includes 10 items

1. About how much time is s(he) allowed to watch TV, videos, or movies each weekday? (total minutes)
2. About how much time is s(he) allowed to watch TV, videos, or movies each weekend day? (total minutes)
3. About how much time is s(he) allowed to play video games each weekday? (total minutes)
4. About how much time is s(he) allowed to play video games each weekend day? (total minutes)
5. I tightly monitor the time my child watches TV or videos during the week (Monday-Friday) (1-5 scale)
6. I tightly monitor the time my child watches TV or videos during the weekend (Saturday-Sunday) (1-5 scale)
7. I tightly monitor the time my child plays video games during the week (Monday-Friday) (1-5 scale)
8. I tightly monitor the time my child plays video games during the weekend (Saturday-Sunday) (1-5 scale)
9. I have control over how much TV my child watches (1-5 scale)
10. I am in charge of how much TV my child watches during his/her free time at home (1-5 scale)

Use of screen time to reward/control child behavior; include 4 items

1. How often do you offer TV, video, or movie time to your child as a reward for good behavior? (1-6 scale)
2. How often do you take away TV, video, or movie time to your child as a reward for bad behavior? (1-6 scale)
3. How often does your child get extra TV, video or movie time as a reward? (1-6 scale)
4. How often do you use TV time to control your child's behavior? Example "If you don't stop that you will not be able to watch TV today". (1-6 scale)

Exposure to TV; includes 3 items

1. How often is the TV in your house on when people are at home? (1-6 scale)
2. How many days per week does your family have the television on during breakfast? (0-7 scale)
3. How many days per week does your family have the television on during the evening meal? (0-7 scale)

Explicit modeling and enjoyment of screen time; includes 6 items:

1. I enjoy watching TV/movies with my child. (1-5 scale)
2. How much do you enjoy watching TV or movies during your free time (1-4 scale)
3. During a typical week, how often do you watch TV or videos with your child? (1-6 scale)
4. During a typical week, how often do you turn on the TV, a video, or movie for your child when the weather is bad? For example, raining, too hot, too cold) (1-6 scale)
5. During a typical week, how often does your child see you watching TV or movies? (1-6 scale)
6. During a typical week, how often do you turn on the TV, a video, or movie for your child so you can get things done around the house? (1-6 response)
